# Investigating the relationship between mental health, resilience and self-compassion among Greek unemployed people

**DOI:** 10.1192/j.eurpsy.2022.1599

**Published:** 2022-09-01

**Authors:** T. Paralikas, K. Vagiatis, M. Gouva, M. Malliarou, S. Kotrotsiou, D. Theofanidis, E. Kotrotsiou

**Affiliations:** 1 UNIVERSITY OF THESSALY, General Department-program Of Nursing Studies, LARISSA, Greece; 2 UNIVERSITY OF THESSALY, Postgraduate Studies In Mental Health, LARISSA, Greece; 3 UNIVERSITY OF IOANNINA, Nursing, IOANNINA, Greece; 4 UNIVERSITY OF THESSALY, Nursing, LARISSA, Greece; 5 UNIVERSITY OF THESSALY, General Department Program Of Nursing Studies, LARISSA, Greece; 6 INTERNATIONAL HELLENIC UNIVERSITY, Nursing, SINDOS THESSALONIKI, Greece

**Keywords:** self-compassion, resilience, mental health, unemployment

## Abstract

**Introduction:**

Unemployment is considered to be one of the most stressful life events that a person may experience. There are a plethora of studies that highlighted the negative effects of unemployment on people’s overall mental health and well-being. Yet, psychological resilience and self-compassion contribute positively in coping with stressful situations and seem to be particularly supportive mechanisms when one is confronted with unemployment.

**Objectives:**

This study intended to investigate the relationships between resilience, self-compassion and mental health in Greek unemployed people and the contribution of specific sociodemographic characteristics in this ‘equation’.

**Methods:**

The study followed a survey design where a sample of 345 Greek unemployed participants completed an online questionnaire, examining the variables under study.

**Results:**

According to the findings, people who reported being unemployed for more than six months showed decreased levels of mental health. Also, the unemployed with higher levels of resilience and self-compassion reported statistically significant higher levels of mental health and vice versa. Finally, self-compassion and psychological resilience were found to be statistically positive related to each other and are predictive factors of mental health with which they are statistically negative related.

**Conclusions:**

The results of this study may contribute to the implementation of interventions aiming at improving mental health and the overall well-being of people affected by long-term unemployment.

**Disclosure:**

No significant relationships.

